# Brown rats and house mice eavesdrop on each other’s volatile sex pheromone components

**DOI:** 10.1038/s41598-020-74820-4

**Published:** 2020-10-19

**Authors:** Elana Varner, Hanna Jackson, Manveer Mahal, Stephen Takács, Regine Gries, Gerhard Gries

**Affiliations:** Department of Biological Sciences, Simon Fraser University, Burnaby, BC V5A 1S6 Canada

**Keywords:** Ecology, Behavioural ecology, Invasive species

## Abstract

Mammalian pheromones often linger in the environment and thus are particularly susceptible to interceptive eavesdropping, commonly understood as a one-way dyadic interaction, where prey sense and respond to the scent of a predator. Here, we tested the “counterespionage” hypothesis that predator and prey co-opt each other’s pheromone as a cue to locate prey or evade predation. We worked with wild brown rats (predator of mice) and wild house mice (prey of brown rats) as model species, testing their responses to pheromone-baited traps at infested field sites. The treatment trap in each of two trap pairs per replicate received sex attractant pheromone components (including testosterone) of male mice or male rats, whereas corresponding control traps received only testosterone, a pheromone component shared between mouse and rat males. Trap pairs disseminating male rat pheromone components captured 3.05 times fewer mice than trap pairs disseminating male mouse pheromone components, and no female mice were captured in rat pheromone-baited traps, indicating predator aversion. Indiscriminate captures of rats in trap pairs disseminating male rat or male mouse pheromone components, and fewer captures of rats in male mouse pheromone traps than in (testosterone-only) control traps indicate that rats do eavesdrop on the male mouse sex pheromone but do not exploit the information for mouse prey location. The counterespionage hypothesis is supported by trap catch data of both mice and rats but only the mice data are in keeping with our predictions for motive of the counterespionage.

## Introduction

Functional roles of mammalian pheromones have routinely been investigated in an intraspecific context, such as territorial marking, sexual signaling and health status conveyance^1^. Yet, closely related species in mammalian communities often use similar communication signals^2^ which facilitates bi-directional (interspecific) olfactory communication^3^ and lowers the relative cost of maintaining sensory receptors^4^. This concept appears to apply to olfactory communication signals of sympatric murine rodents, including the brown rat, *Rattus norvegicus,* and the house mouse, *Mus musculus,* because there is overlap in pheromone components of female mice and female rats^5,6^. Native to the plains of Asia^7,8^, brown rats and house mice co-evolved in a predator–prey relationship, with rats preying on mice^9,10^. Both of these macrosmatic rodents are prolific scent markers^11,12^ that rely on their sense of smell during mostly nocturnal activity bouts. Within each species, respective urine marks offer a wealth of information about the signaler, including its age^13^, health^14^, breeding status^15,16^, dominance^17^, kinship and individual identity^18,19^. Moreover, rat odor elicits an innate avoidance behavior in mice^20,21^.

Urine marks of rats and mice also disseminate sex attractant pheromone components. Although rats and mice share some pheromone components (e.g., testosterone, progesterone, estradiol)^22^, the more volatile sex attractant pheromone components of males differ markedly. The ketone blend in urine marks of male brown rats (2-heptanone, 4-heptanone, 3-ethyl-2-heptanone, 2-octanone, 2-nonanone, 4-nonanone^6^) bears no resemblance to pheromone components emanating from urine marks of male house mice (3,4-dehydro-*exo*-brevicomin; 2-*sec*-butyl-4,5-dihydrothiazole^23,24^).

While acoustic and visual signals have a fleeting presence, odors and specifically pheromones often linger in the environment^25,26^. This makes pheromones particularly susceptible to inter-species exploitation^12,26,27^ which is well known in insects^28–31^ but has hardly been studied in mammals^4,32–35^. Studies on mammalian prey eavesdropping on the communication of their predators have focused on audio and visual communication signals^36,37^. Only two studies have demonstrated that rodents recognize the presence of predators based on their major urinary proteins and lacrimal proteins^10,38^. Compared to these high molecular-weight proteins, volatile sex attractant pheromone components contrive long-range mate attraction^39^ and thus are particularly susceptible to interspecies-eavesdropping^36^.

Intercepting scent communication in vertebrate communities has long been studied, or viewed, as a one-way dyadic interaction, with prey sensing predator scent^25^. For instance, feline and canine odors elicit stereotyped fear and avoidance responses in rodents^34^. However, expanded views of auditory and visual communication systems now portray a multi-directional eavesdropper community network^25,36^. For example, mustelid, canid and felid predators exploit mammalian prey scent to locate prey^12,40^, imposing significant costs on chemical signaling in the prey species^41–43^. Whether vertebrate predator–prey interactions are informed and guided by bi-directional (mutual) eavesdropping, or “counterespionage”, on scent signals is entirely unknown, as are the underlying mechanisms.

Scent marks disseminate a myriad of odorants, only a few of which are pheromones, and hardly any pheromones are known to date. When prey avoided locations scent-marked by predators^20,34,44^, and predators responded to scent marks of prey^12^, these animals may simply have recognized generic prey and predator scent without necessarily eavesdropping on pheromone signals of target prey or predator foe. Testing the concept of mutual eavesdropping by predator and prey on each other’s pheromones is contingent upon pheromone identification and the availability of synthetic pheromone. When synthetic volatile sex attractant pheromone components of both brown rats (predator of mice^9^) and house mice (prey of rats^10^) became available^6,22–24,45,46^, the stage was set for testing the counterespionage hypothesis that mice co-opt rat pheromone components as cues to avoid rat predation, and rats co-opt mouse pheromone components as cues to facilitate mouse prey location. Testing these hypotheses, we were cognizant that the natural sex pheromone of mice and rats comprises additional constituents (e.g., urinal and lacrimal proteins^10,38^) which—expense-wise—could not be included in our synthetic pheromone lure, and that these constituents as well as non-pheromonal odors^47^ may amplify any counterespionage evidence demonstrated in our study.

## Results

### Hypothesis 1: mice co-opt rat pheromone as a cue to avoid rat predation

**Figure 1:**
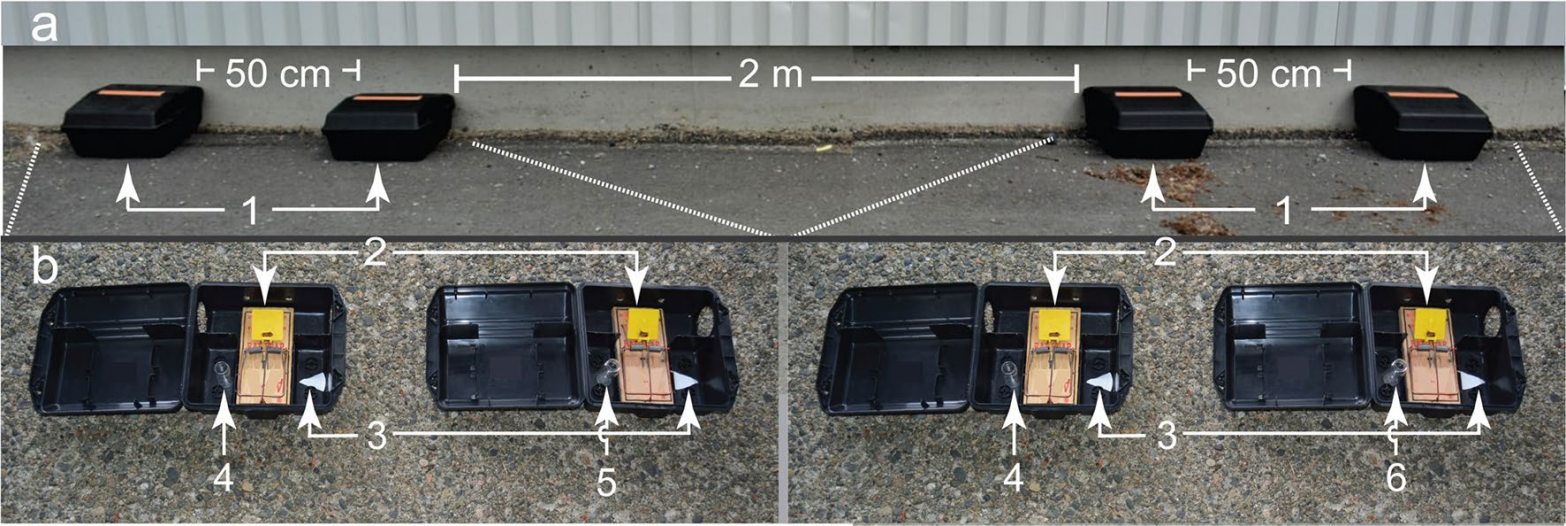
Photographs illustrating (**a**) the double-set, paired trap box design of an experimental replicate, and (**b**) details of snap trap, food bait and pheromone lure. Each experimental replicate (n = 157) consisted of two pairs of large trap boxes (placed in rat-infested sites), or two pairs of small trap boxes (placed in mouse-infested sites; not shown in this figure), for capturing rats and mice, respectively, with 0.5-m spacing between the boxes in each pair, and at least 2 m between pairs. Numbers refer to: 1 = trap box; 2 = snap trap with food bait^69^ for capturing (killing) responding rodents; 3 = filter paper treated with synthetic testosterone (a pheromone component shared by male brown rats and male house mice); 4–6 = a 20-ml glass scintillation vial containing plain mineral oil (4; control stimulus) or mineral oil laced with sex attractant pheromone components of either male house mice (5) or male brown rats (6). Note: the smaller trap boxes for mice (not shown here) were fitted with glass scintillation vials reduced in height (cut to size).

**Figure 2:**
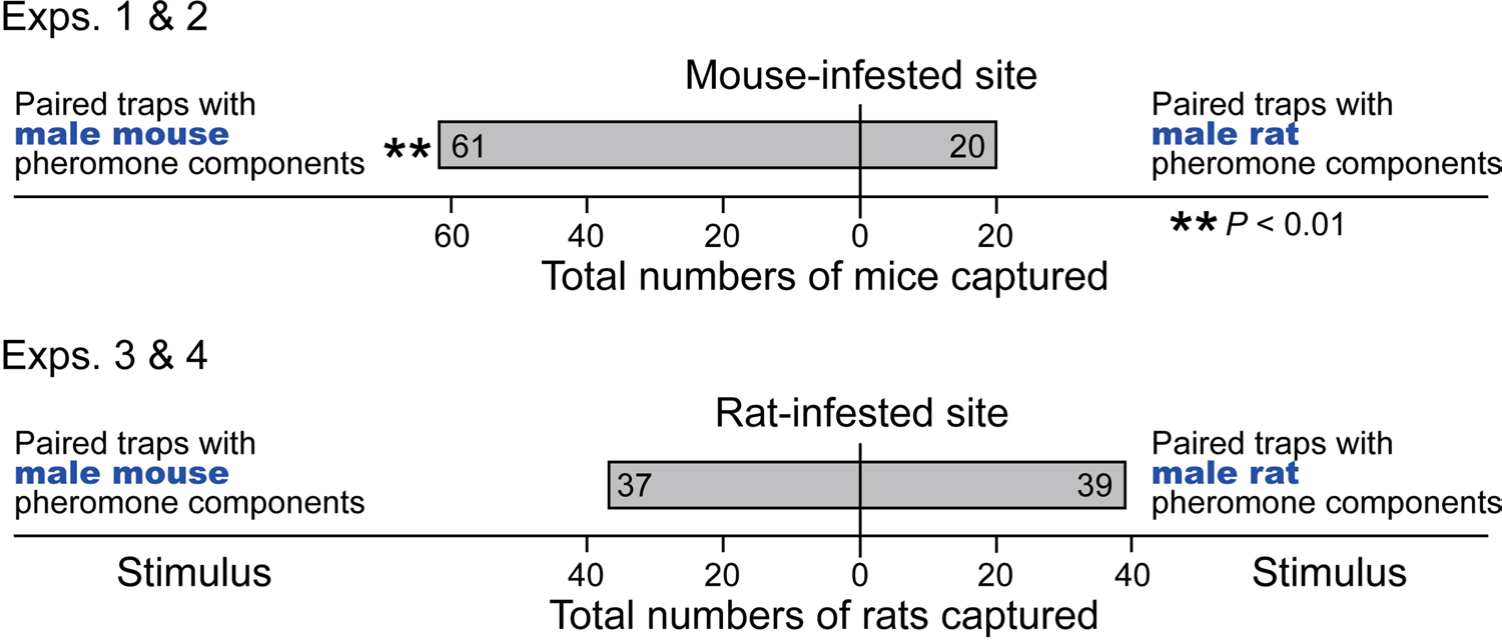
Trap catch data revealing that house mice are averse to macro-locations (trap box pairs; see Fig. 1) indicative of brown rat presence. The treatment trap in each pair received the volatile synthetic sex attractant pheromone components of male house mice (testosterone, 3,4-dehydro-*exo*-brevicomin, 2-*sec*-butyl-4,5-dihydrothiazole) or brown rats (testosterone, 2-heptanone, 4-heptanone, 3-ethyl-2-heptanone, 2-octanone, 2-nonanone, 4-nonanone), whereas corresponding control trap boxes received testosterone only. Trap pair locations with rat pheromone components captured 3.05 times fewer mice than trap pair locations with mouse pheromone components, whereas trap pair locations with rat or mouse pheromone components captured equal numbers of rats, revealing predator-aversion behavior by mice and no evidence for prey-seeking behavior by rats. The asterisks indicate a significant difference in the number of mice captured in paired traps (χ^2^-tests with Yate’s correction for continuity compared against a theoretical 50:50 distribution, ** *P* < 0.01).

**Figure 3:**
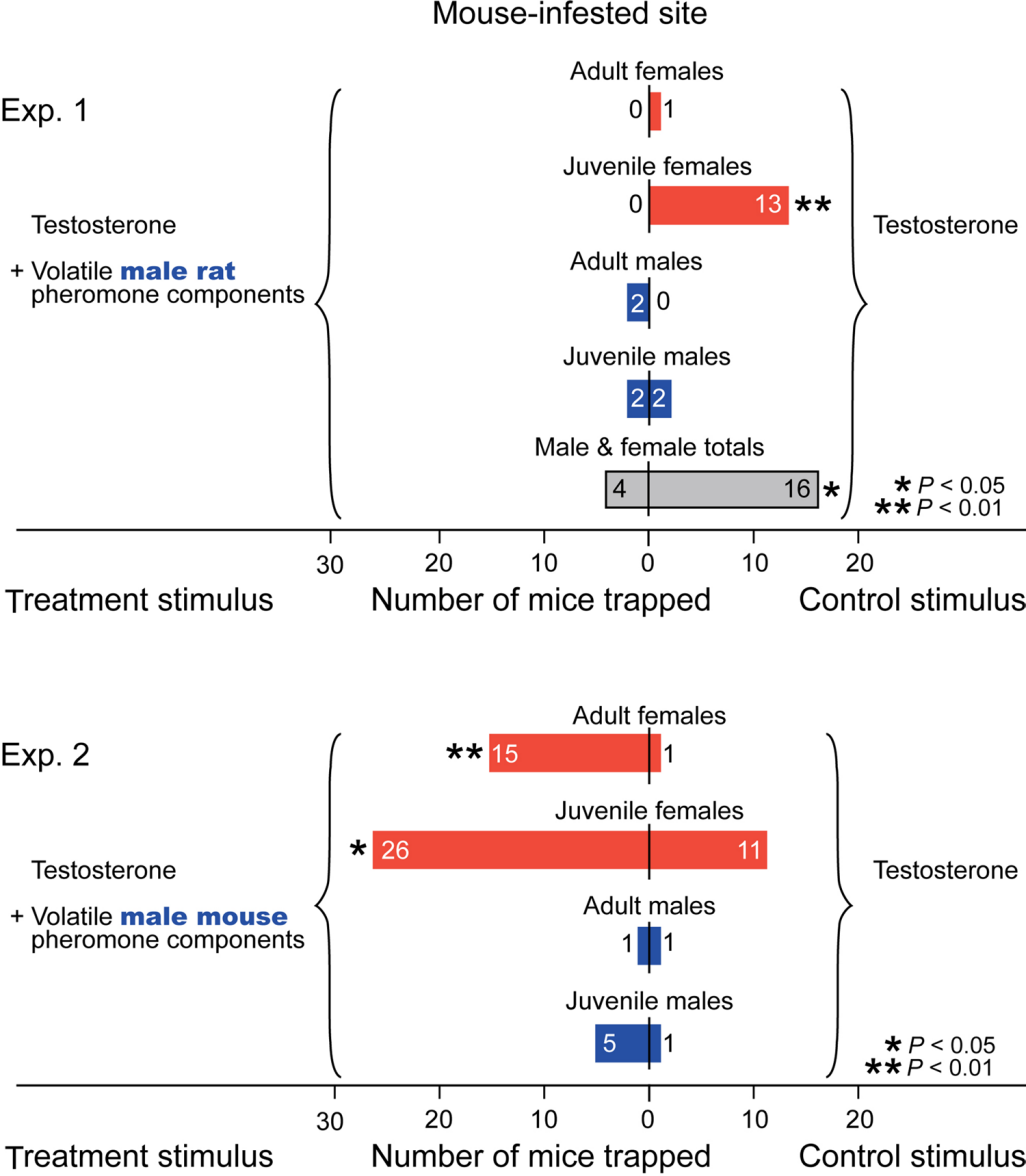
Trap catch data revealing that female house mice stay away from and seek micro-locations (specific trap boxes) indicative of male brown rat and male house mouse presence, respectively. The treatment trap in each pair received the volatile synthetic sex attractant pheromone components of male house mice (testosterone, 3,4-dehydro-*exo*-brevicomin, 2-*sec*-butyl-4,5-dihydrothiazole) or male brown rats (testosterone, 2-heptanone, 4-heptanone, 3-ethyl-2-heptanone, 2-octanone, 2-nonanone, 4-nonanone), whereas corresponding control trap boxes received testosterone only. The asterisks indicate a significant difference in the number of mice captured in treatment and control traps (χ^2^-tests with Yate’s correction for continuity compared against a theoretical 50:50 distribution; * *P* < 0.05, ** *P* < 0.01).

In mouse-infested sites, trap pairs (see Fig. 1 for the general experimental design) baited with synthetic sex pheromone components of male rats captured 3.05 times fewer mice than trap pairs baited with synthetic pheromone components of male mice (χ^2^ = 19.75, *P* < 0.0001) (Fig. 2, top), suggesting that mice avoided macro-locations indicative of rat presence. Moreover, traps baited with male mouse pheromone components captured 15-times more adult female mice and 2.4-times more juvenile female mice than control traps baited with testosterone alone (adult females: χ^2^ = 10.56, *P* < 0.01; juvenile females: χ^2^ = 5.30, *P* < 0.05) (Fig. 3, bottom), confirming a synergistic effect of testosterone, brevicomin and thiazole on attraction of female mice^22^. Captures of adult male mice (2) and juvenile male mice (6) were insufficient to warrant statistical analysis. Conversely, traps baited with male rat pheromone components failed to capture a single female mouse, whereas corresponding (testosterone-only) control traps captured one adult female mouse and 13 juvenile female mice (χ^2^ = 11.01, *P* < 0.01) (Fig. 3, top), further indicating recognition and avoidance of micro-locations indicative of rat presence. Captures of adult male mice (2) and juvenile male mice (4) in traps baited with male rat lures were insufficient for statistical analyses.

### Hypothesis 2: rats co-opt mouse pheromone as a cue to facilitate mouse-prey location

**Figure 4:**
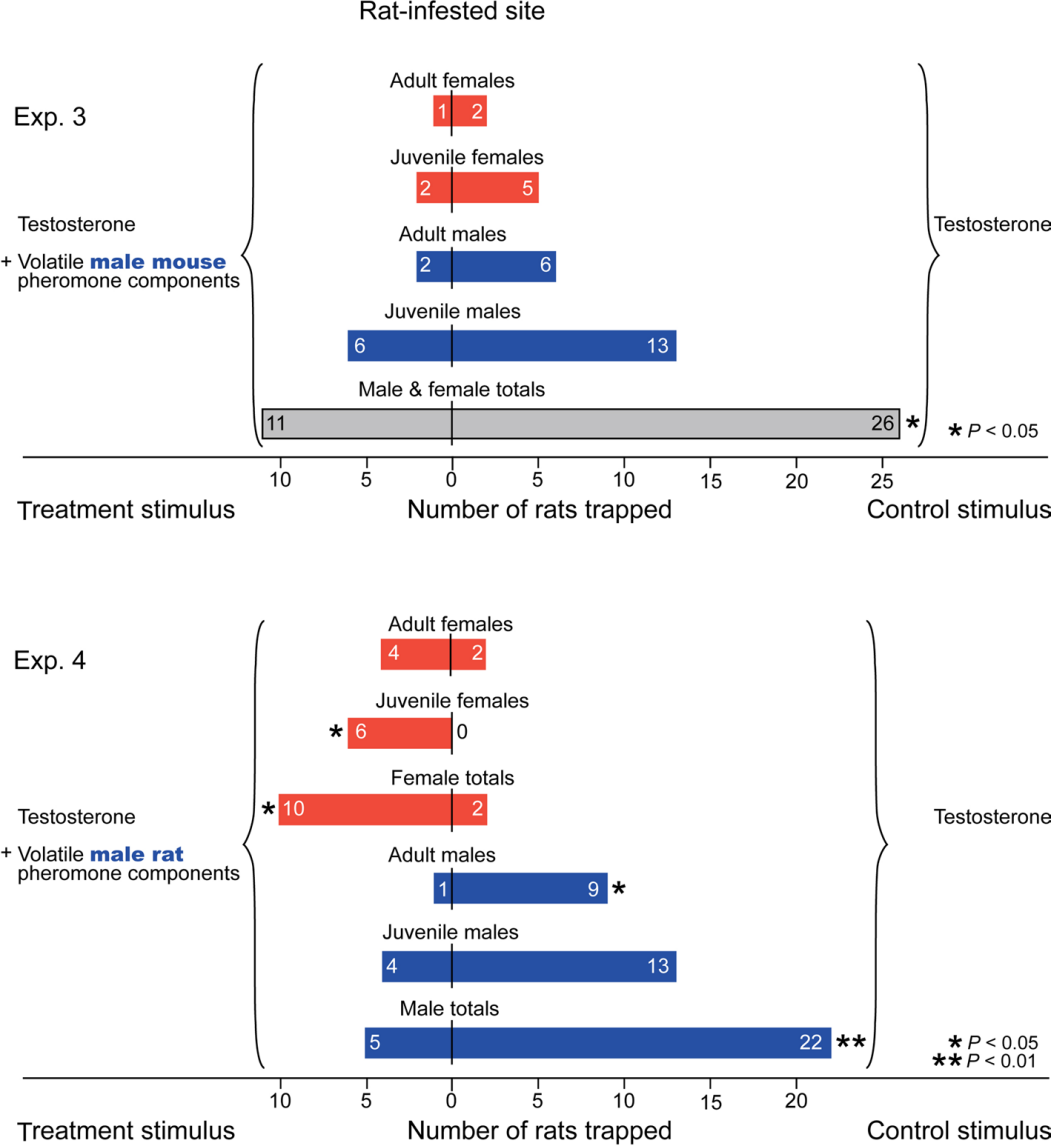
Trap catch data revealing that brown rats stay away from micro-locations (specific trap boxes) indicative of male mouse presence, and that female and male brown rats seek and avoid micro-locations indicative of prospective mates and rival males, respectively. The treatment trap in each pair received the volatile synthetic sex attractant pheromone components of male house mice (testosterone, 3,4-dehydro-*exo*-brevicomin, 2-*sec*-butyl-4,5-dihydrothiazole) or male brown rats (testosterone, 2-heptanone, 4-heptanone, 3-ethyl-2-heptanone, 2-octanone, 2-nonanone, 4-nonanone), whereas corresponding control trap boxes received testosterone only. The asterisks indicate a significant difference in the number of rats captured in treatment and control traps (χ^2^-tests with Yate’s correction for continuity compared against a theoretical 50:50 distribution; * *P* < 0.05, ** *P* < 0.01).

In rat-infested sites, trap pairs baited with synthetic male mouse pheromone components captured as many rats as trap pairs baited with synthetic male rat pheromone components (χ^2^ = 0.01, *P* > 0.05) (Fig. 2, bottom), revealing that foraging rats did not actively seek macro-locations indicative of mouse prey. On the contrary, traps baited with male mouse pheromone captured significantly fewer male and female rats than (testosterone-only) control traps (χ^2^ = 5.30, *P* < 0.05) (Fig. 4, top). Traps baited with a blend of male rat pheromone components—expectedly—captured significantly more females and significantly fewer males than (testosterone-only) control traps (females: χ^2^ = 4.08, *P* < 0.05; males: χ^2^ = 9.48, *P* < 0.01) (Fig. 4, bottom), confirming the reported attractiveness and deterrence of male rat pheromone components to female and male rats, respectively^6^.

## Discussion

Our data support the “counterespionage” hypothesis. Mice and rats did eavesdrop on each other’s sex pheromone but they used the information they gleaned in a way only partially in keeping with our predictions for motive. This is the first evidence for bi-directional interspecific recognition of sex pheromones within a guild of mammals and between mammalian prey and predator. Our data also reveal that the sex attractant pheromone components of male mice (brevicomin and thiazole) and male rats (ketone blend) are underlying mechanisms that impart species-specificity to pheromonal communication between these murine rodents.

We deemed field experiments with pheromone-baited traps the most effective way to test our “counterespionage” hypothesis that predator and prey co-opt each other’s pheromone as a cue to locate prey or evade predation. We considered trap captures of wild male and female mice, and wild male and female rats, an excellent means to reveal attraction or deterrence of these murine rodents to their own pheromone and that of their mouse prey or rat foe. For future studies, however, we plan on video recording the behavior of rats and mice near select trap boxes to (*i*) reveal subtleties of behavioral responses indicative of attraction or fear according to the lure presented, and (*ii*) document the number of rodents that are approaching trap boxes but are not getting captured, indicating the proportion of the population that generates the data. Testing wild rodents in their natural environments was imperative because domesticated rodents in laboratory settings are known to behave differently than their wild counterparts^48–50^. As mice and rats typically do not share the same habitat^51^, we needed to run experiments in locations infested with either mice or rats.

As predicted, female house mice co-opted the sex pheromone of male rats as a cue indicative of rat presence and potential predation risk by rats. Female mice largely avoided locations of paired traps disseminating synthetic male rat pheromone (Fig. 2), and not one single mouse female entered a trap box baited with rat sex pheromone (Fig. 3). These results are not surprising given that predator avoidance behavior is critical to the survival of mice, whereas rats do not avoid the odors of their predators, at least not when collecting food in relatively safe and familiar habitats^52^. Recognizing scent marks of predators such as rats and cats enables mice to detect and avoid locations frequented by these predators, or to adjust their temporal foraging pattern accordingly^53^. Sensory neurons in the vomeronasal organs of mice detect specific major urinary proteins in urine scent marks of rats and cats which ultimately cause avoidance responses by mice^10,54,55^. Similarly, a lacrimal protein of rats (rat CRPI) decreases locomotion of mice and lowers their body temperature and heart rate^38^. However, all behavioral responses by mice in these studies to urinary and lacrimal proteins of rats were recorded in the confines of very small laboratory bioassay arenas where even “heavy” proteins could invoke behavior-modifying effects. Our field data obtained with populations of wild mice and rats conclusively show that the volatile sex attractant pheromone components of male rats have a long-distance aversion effect (Fig. 2) and a short-distance avoidance effect (Fig. 3) on female mice.

The hypothesis that rats co-opt the sex pheromone of male mice as a cue to locate mouse prey was not supported by our data. In rat-infested sites, locations of paired traps disseminating synthetic male mouse pheromone did not yield more captures of foraging rats than locations of paired traps disseminating synthetic male rat pheromone (Fig. 2). Remarkably, both male and female rats recognized the male mouse sex pheromone, and many stayed away from trap boxes, or “burrows”, apparently occupied by a male mouse (Fig. 4). While female rats may have simply recognized the “message” of an inappropriate (heterospecific) mate, the aversion responses of male rats can only be explained in a context other than sexual communication and mate recognition. Irrespective, rats did not exploit male mouse pheromone to locate mouse prey. Rather, they showed the propensity to avoid encounters with potential male mouse prey. There are several explanations for this seemingly peculiar behavior. First, brown rats are omnivores and only opportunistic predators of mice, which are not a primary food source for rats in the urban and industrial settings where we trapped. Second, all of our trapping sites had an abundant and constant supply of food other than live mouse prey, making rats not reliant on predation success for survival. Third (and perhaps least likely), brown rats may have traded the nutritional benefits of a proteinaceous male mouse meal for not risking injury during predation bouts.

Our study has shown that mammalian pheromones, comparable to auditory or visual communication signals, are under surveillance by a network of eavesdroppers. “Designed” for incessant information flow, rodent sex pheromone components and their delivery systems are particularly susceptible to eavesdropping on these signals by illicit recipients, such as predators or prey. Major urinary proteins in urine scent marks of mice and rats serve as dissemination conduits for the volatile sex attractant pheromone components^56–58^. These delivery systems are so sophisticated that they even have inherent timestamps, informing the signal recipient of how recently the message was placed^59^. The functional role of mouse and rat major urinary proteins could not be assessed in our field study, but we surmise that these proteins would have contributed to the behavioral effects prompted by the sex *attractant* pheromone components.

Our findings that brown rats and house mice recognize each other’s sex pheromone engender exciting new research opportunities, particularly in conservation ecology. The long-distance aversion effect of brown rat pheromone components on house mice (Figs. 2, 3) could be used as a means to expel mice from biodiverse hotspots in island communities, where rat control has prompted harmful outbreaks of mice^60^. The tactic of exploiting predator scent for pest control^35,61^ was successful in various wildlife conservation projects^62–64^ but sourcing of scent directly from predators is impractical and would not be necessary if synthetic rat pheromone was used for manipulation of mice. The failure of some studies to achieve repellent effects with predator odors for pest control^34^ has likely multiple reasons, one of which being insuffcient longevity of predator urine or feces odors. Slow-release formulations of synthetic pheromone components, possibly presented in combination with some non-pheromonal predator odors^47^, may not only prolong the effect of predator scent on prey but make this tactic more affordable than sourcing of scent directly from predators.

If synthetic mouse sex pheromones were experimentally shown to attract feral cats, synthetic mouse pheromone lures could be developed for capturing, and subsequent neutering of feral cats that otherwise would continue to reproduce prolifically, extending their already devastating impact on bird populations^65^. The same pheromone lures could be deployed for trapping feral cats that have invaded, or were deliberately introduced to, island communities where they now threaten seabird colonies^66^ and many endemic reptiles^67^. If the eavesdroppers’ network were to include other mesopredators of murine rodents such as the red fox, *Vulpes vulpes*, or striped skunk, *Mephitis mephitis*, then synthetic rodent pheromone could be used to help eliminate diseases from mesopredator populations. For example, adding synthetic rodent pheromone to baits laced with oral rabies vaccine^68^ would likely make these baits olfactorily more apparent to foraging predators and thus expedite bait location and disease elimination.

## Materials and methods

### General design of field experiments

Parallel field experiments for trapping house mice and brown rats were run between March–June 2017 and October 2016–November 2019 in mouse-infested sites (Exps. 1, 2; 81 paired trap boxes each for mice and rats) and in rat-infested sites (Exps. 3, 4; 76 paired trap boxes each for mice and rats) in the Fraser Valley of British Columbia, Canada. The four sites infested with rats (inferred by the presence of 0.6- to 1.3-cm long fecal pellets with pointed ends) included a food production facility, a food bank, and two recycling centers, whereas the two sites infested with mice (inferred by the presence of 0.6-cm long fecal pellets with blunt ends) included a duck farm and a bird sanctuary. Based on fecal pellet evidence, all sites were exclusively infested with either rats or mice. Population densities in these sites were likely weak to moderate based on infrequent rodent sightings, the amount of feces present, and the time needed to generate the trap catch data. In all sites, rodents had steady access to animal or human food and were exposed to predation by feral cats and owls. Mouse-infested sites had been used in previous research projects with mice^5,22,46,69^ but were not used for one year prior to the onset of our study. All sites were subject to rodent control measures mainly in the form of poison bait stations.

In each site, experimental replicates for mice and rats were set up along interior or exterior walls of buildings (Fig. 1). Each replicate consisted of two sets of paired trap boxes (PROTECTA Mouse or Rat, Bell Laboratories Inc., Madison, WI 53,704, USA), with 0.5-m spacing between the boxes in each pair, and at least 2 m between pairs (Fig. 1). Each trap box contained a Victor snap trap (M325 M7 Pro mouse or M326 M7 Pro rat Woodstream Co., Lititz, PA 175,543, USA) that was set with a food bait^69^ which prompted feeding and thus capture of responding mice or rats. Twice or 3-times every week, traps were checked, and food baits and pheromone lures (see below) replaced. Captured rodents were assessed for their age (juvenile or adult) based on genitalia development^70^, and for their sex based on ano-genital distance^71^ or PCR genotyping carried out on DNA extracted from ear or tail clips^72^. Whenever a mouse or a rat had been captured, a new trap box and snap trap were deployed. This procedure ensured that the odor of captured mice or rats did not affect future captures. The position of the treatment and the control trap box within a trap box pair was re-randomized after each capture. The research protocol was approved and supported by the Animal Care Committee of Simon Fraser University (protocol #1159B-15 and #1295B-19) which abides by the Canadian Council on Animal Care guidelines.

### Synthetic sex pheromone components tested

Both the treatment and the control trap box in each trap box pair received testosterone, a pheromone component of low volatility shared between house mouse and brown rat males^22^. Adding the volatile sex attractant pheromone components of either male mice or male rats (see below) to testosterone, we could then test whether these components impart species-specificity to the sex pheromone blend and enable cross-recognition of predator or prey communication signals. This plain experimental design was guided by recent studies already showing that: (1) synthetic testosterone on its own tested *versus* an unbaited control strongly attracts female mice and female rats^22^; (2) traps baited with synthetic sex attractant pheromone components of male mice (brevicomin & thiazole; see below), or of male rats (ketone blend; see below), capture significantly more female mice^46^, and more female rats^6^, than unbaited control traps; and (3) synthetic trap lures containing both testosterone (or androstenone) and sex attractant pheromone components of male rats or male mice synergistically attract more female rats^22^, and more female mice^22,73^, than partial pheromone lures containing either the sex steroid or the sex attractant pheromone components. As the more complete pheromone lure for mice and rats is clearly more effective than partial pheromone lures, there is no need for testing it further *versus* unbaited controls.

Testosterone was dissolved in acetonitrile (50 μl) and applied to a piece of filter paper at the biologically relevant dose of 750 ng (about five times the amount of testosterone a single male mouse discharged with urine during one day)^22^. The treatment box in each pair received synthetic sex attractant pheromone components of either male house mice [3,4-dehydro-*exo*-7-ethyl-5-methyl-6.8-dioxabicyclo[3.2.1]octane (= 3,4-dehydro-*exo*-brevicomin = brevicomin); 2-*sec*-butyl-4,5-dihydrothiazole (= thiazole)] or male brown rats (2-heptanone, 4-heptanone, 3-ethyl-2-heptanone, 2-octanone, 2-nonanone, 4-nonanone). The house mouse pheromone components brevicomin and thiazole were each formulated at 1 mg in mineral oil (10 ml) and contained in a 20-ml glass scintillation vial (VWR International, LLC Randor, PA 19,087, USA). This formulation afforded the release of brevicomin and thiazole at rates of 180 ng h^−1^ and 75 ng h^−1^, respectively, very similar to the hourly release rates of these two compounds from bedding material soiled by laboratory-kept male mice^46^. The sex attractant pheromone components of male brown rats were formulated as a 1-mg blend at the same ratio [2-heptanone (100), 4-heptanone (10), 3-ethyl-2-heptanone (10), 2-octanone (1), 2-nonanone (1), 4-nonanone (10)]) as found in headspace odorants of male rat urine, and afforded release rates comparable to those from soiled bedding material of laboratory-kept rats^6^. The potential of glassware or mineral oil to modulate the effects of brevicomin and thiazole or the blend of ketones was minimized by fitting treatment and control trap boxes in each trap pair with the same glassware and volume of mineral oil.

### Statistical analyses

We analyzed all data with R 3.5.0^74^. For each of experiments 1–4, we compared the proportion of captures in treatment and control traps against a theoretical 50:50 distribution, using a χ^2^-test with Yate’s correction for continuity. We also used paired χ^2^-tests to compare total captures of mice and of rats in traps baited with synthetic pheromone components of male mice or male rats in mouse- and rat-infested sites.

## Data Availability

All data are presented in the main body of the manuscript.

## References

[1] Wyatt TD (2013). Pheromones and Animal Behavior.

[2] Hughes NK, Korpimäki E, Banks PB (2010). The predation risks of interspecific eavesdropping: weasel-vole interactions. Oikos.

[3] Garvey PM (2017). Exploiting interspecific olfactory communication to monitor predators. Ecol. Appl..

[4] Parsons MH (2018). Biologically meaningful scents: a framework for understanding predator–prey research across disciplines. Biol. Rev..

[5] Varner E, Gries R, Takács S, Fan S, Gries G (2019). Identification and field testing of volatile components in the sex attractant pheromone blend of female house mice. J. Chem. Ecol..

[6] Takács S, Gries R, Zhai H, Gries G (2016). The sex attractant pheromone of male brown rats: identification and field experiment. Angew. Chemie Int. Ed..

[7] Din W (1996). Origin and radiation of the house mouse: clues from nuclear genes. J. Evol. Biol..

[8] Puckett EE (2016). Global population divergence and admixture of the brown rat (Rattusnorvegicus). Proc. R. Soc. B Biol. Sci..

[9] Karli P (1956). The Norway rat’s killing response to the white mouse: an experimental analysis. Source Behav..

[10] Papes F, Logan DW, Stowers L (2010). The vomeronasal organ mediates interspecies defensive behaviors through detection of protein pheromone homologs. Cell.

[11] Slotnick B (2001). Animal cognition and the rat olfactory system. Med. J. Aust..

[12] Hughes NK, Price CJ, Banks PB (2010). Predators are attracted to the olfactory signals of prey. PLoS ONE.

[13] Osada K, Tashiro T, Mori K, Izumi H (2008). The identification of attractive volatiles in aged male mouse urine. Chem. Senses.

[14] Kavaliers M, Choleris E, Pfaff DW (2005). Recognition and avoidance of the odors of parasitized conspecifics and predators: differential genomic correlates. Neurosci. Biobehav. Rev..

[15] Hurst JL (1989). The complex network of olfactory communication in populations of wild house mice *Mus domesticus* rutty: urine marking and investigation within family groups. Anim. Behav..

[16] Mossman CA, Drickamer LC (1996). Odor preferences of female house mice (*Mus domesticus*) in seminatural enclosures. J. Comp. Psychol..

[17] Jones RB, Nowell NW (1973). Aversive and aggression-promoting properties of urine from dominant and subordinate male mice. Anim. Learn. Behav..

[18] Barnard CJ, Fitzsimons J (1988). Kin recognition and mate choice in mice: the effects of kinship, familiarity and social interference on intersexual interaction. Anim. Behav..

[19] He J, Ma L, Kim S, Nakai J, Yu CRR (2008). Encoding gender and individual information in the mouse vomeronasal organ. Science.

[20] Yang M (2004). The rat exposure test: a model of mouse defensive behaviors. Physiol. Behav..

[21] Amaral VCS, Santos Gomes K, Nunes-de-Souza RL (2010). Increasedcorticosterone levels in mice subjected to the rat exposure test. Horm. Behav..

[22] Takács S, Gries R, Gries G (2017). Sex hormones function as sex attractant pheromones in house mice and brown rats. ChemBioChem.

[23] Jemiolo B, Alberts J, Sochinski-Wiggins S, Harvey S, Novotny M (1985). Behavioural and endocrine responses of female mice to synthetic analogues of volatile compounds in male urine. Anim. Behav..

[24] Novotny M (1985). Synthetic pheromones that promote inter-male aggression in mice. Proc. Natl. Acad. Sci. USA.

[25] Banks PB, Daly A, Bytheway JP (2016). Predator odours attract other predators, creating an olfactory web of information. Biol. Lett..

[26] Roitberg BD, Córdoba-Aguilar A, González-Tokman D, González-Santoyo I (2018). Chemical communication. Insect Behavior: From Mechanisms to Ecological and Evolutionary Consequences.

[27] Vasudevan A, Vyas A (2013). Kairomonal communication in mice is concentration-dependent with a proportional discrimination threshold. F1000Research.

[28] Danci A, Schaefer PW, Schopf A, Gries G (2006). Species-specific close-range sexual communication systems prevent cross-attraction in three species of Glyptapanteles parasitic wasps (*Hymenoptera: Braconidae*). Biol. Control.

[29] Wen X-LL, Wen P, Dahlsjö C, Sillam-Dussès D, Šobotník J (2017). Breaking the cipher: ant eavesdropping on the variational trail pheromone of its termite prey. Proc. R. Soc. B Biol. Sci..

[30] Haynes KF, Yeargan KV (1999). Exploitation of intraspecific communication systems: illicit signalers and receivers. Ann. Entomol. Soc. Am.

[31] Dong S (2018). Olfactory eavesdropping of predator alarm pheromone by sympatric but not allopatric prey. Anim. Behav..

[32] Sbarbati A, Osculati F (2006). Allelochemical communication in vertebrates: kairomones, allomones and synomones. Cells Tissues Organs.

[33] Apps, P., Rafiq, K. & McNutt, J. W. Do carnivores have a world wide web of interspecific scent signals? in *Chemical Signals in Vertebrates* (ed. Buesching, C. D.) 182–202 (2019).

[34] Apfelbach R, Blanchard CD, Blanchard RJ, Hayes RA, Mcgregor IS (2005). The effects of predator odors in mammalian prey species: a review of field and laboratory studies. Neurosci. Biobehav. Rev..

[35] Jones ME (2016). A nose for death: Integrating trophic and informational networks for conservation and management. Front. Ecol. Evol..

[36] McGregor PK, Larry RS (2009). Communication networks and eavesdropping in animals. Encyclopedia of Neuroscience.

[37] Peake, T. M. Eavesdropping in communication networks. in *Animal Communication Networks* (ed. McGregor, P. K.) 13–37 (2005).

[38] Tsunoda M (2018). Identification of an intra- and inter-specific tear protein signal in rodents. Curr. Biol..

[39] Ardeh MJ, De Jong PWD, Loomans AJM, Van Lenteren JC (2004). Inter- and intraspecific effects of volatile and nonvolatile sex pheromones on males, mating behavior, and hybridization in *Eretmocerus mundus* and *E. eremicus* (Hymenoptera: Aphelinidae). J. Insect Behav..

[40] Ylönen H, Sundell J, Tiilikainen R, Eccard JA, Horne T (2003). Weasels’ *(Mustela nivalis nivalis)* preference for olfactory cues of the vole *(Clethrionomys glareolus)*. Ecology.

[41] Zhang Y-HH, Liang H-CC, Guo H-LL, Zhang J-XX (2016). Exaggerated male pheromones in rats may increase predation cost. Curr. Zool..

[42] Hughes NK, Kelley JL, Banks PB (2009). Receiving behaviour is sensitive to risks from eavesdropping predators. Oecologia.

[43] Koivula M, Korpimäki E, Korpimaki E (2001). Do scent marks increase predation risk of microtine rodents?. Oikos.

[44] May MD, Bowen MT, Mcgregor IS, Timberlake W (2012). Rubbings deposited by cats elicit defensive behavior in rats. Physiol. Behav..

[45] Schwende FJ, Wiesler D, Jorgenson JW, Carmack M, Novotny M (1986). Urinary volatile consituents of the house mouse, *Mus musculus*, and their endocrine dependency. J. Chem. Ecol..

[46] Musso AE, Gries R, Zhai H, Takács S, Gries G (2017). Effect of male house mouse pheromone components on behavioral responses of mice in laboratory and field experiments. J. Chem. Ecol..

[47] Ferrero DM (2011). Detection and avoidance of a carnivore odor by prey. PNAS.

[48] Wolff JO (2003). Laboratory studies with rodents: facts or artifacts?. Bioscience.

[49] Calisi RM, Bentley GE (2009). Lab and field experiments: are they the same animal?. Horm. Behav..

[50] Kondrakiewicz K, Kostecki M, Szadzińska W, Knapska E (2019). Ecological validity of social interaction tests in rats and mice. Genes Brain Behav..

[51] de Masi E, Vilaça P, TerezaPepeRazzolini M (2009). Environmental conditions and rodent infestation in Campo Limpo district, São Paulo municipality, Brazil. Int. J. Environ. Health Res..

[52] Stryjek R, Mioduszewska B, Spaltabaka-Gędek E, Juszczak GR (2018). Wild norway rats do not avoid predator scents when collecting food in a familiar habitat: a field study. Sci. Rep..

[53] Lima SL, Bednekoff PA (1999). Temporal variation in danger drives antipredatorbehavior: the predation risk allocation hypothesis. Am. Nat..

[54] Pérez-Gómez A (2015). Innate predator odor aversion driven by parallel olfactory subsystems that converge in the ventromedial hypothalamus. Curr. Biol..

[55] Isogai Y (2011). Molecular organization of vomeronasal chemoreception. Nature.

[56] Bacchini A, Gaetani E, Cavaggioni A (1992). Pheromone binding proteins of the mouse, Musmusculus. Experientia.

[57] Novotny MV, Ma W, Wiesler D, Žídek L (1999). Positive identification of the puberty-accelerating pheromone of the house mouse: the volatile ligands associating with the major urinary protein. Proc. R. Soc. B Biol. Sci..

[58] Hurst J, Robertson D, Tolladay U, Beynon R (1998). Proteins in urine scent marks of male house mice extend the longevity of olfactory signals. Anim. Behav..

[59] Beynon RJ, Hurst JL (2004). Urinary proteins and the modulation of chemical scents in mice and rats. Peptides.

[60] Caut S (2007). Rats dying for mice: modelling the competitor release effect. Austral. Ecol..

[61] Campbell-Palmer R, Rosell F (2011). The importance of chemical communication studies to mammalian conservation biology: a review. Biol. Conserv..

[62] Sparrow EE, Parsons MH, Blumstein DT (2016). Novel use for a predator scent: preliminary data suggest that wombats avoid recolonising collapsed burrows following application of dingo scent. Aust. J. Zool..

[63] Friesen MR, Beggs JR, Gaskett AC (2017). Sensory-based conservation of seabirds: a review of management strategies and animal behaviours that facilitate success. Biol. Rev..

[64] Campbell-Palmer R, Rosell F (2010). Conservation of the Eurasian beaver Castor fiber: an olfactory perspective. Mamm. Rev..

[65] Loss SR, Will T, Marra PP (2013). The impact of free-ranging domestic cats on wildlife of the United States. Nat. Commun..

[66] BirdLife International. *State of the world’s birds: taking the pulse of the planet*. (2018).

[67] Courchamp F, Langlais M, Sugihara G (1999). Cats protecting birds: modelling the mesopredator release effect. J. Anim. Ecol..

[68] MacInnes CD (2001). Elimination of rabies from red foxes in eastern Ontario. J. Wildl. Dis..

[69] Takács S (2017). New food baits for trapping house mice, black rats and brown rats. Appl. Anim. Behav. Sci..

[70] Safranski TJ, Lamberson WR, Keisler DH (1993). Correlations among three measures of puberty in mice and relationships with estradiol concentration and ovulation. Biol. Reprod..

[71] Schneider JE, Wysocki CJ, Nyby J, Whitney G (1978). Determining the sex of neonatal mice (*Mus musculus*). Behav. Res. Methods Instrum..

[72] Dhakal P, Soares MJ (2017). Single-step PCR-based genetic sex determination of rat tissues and cells. Biotechniques.

[73] Varner, E, Gries, R. & Gries, G. Attractant blend composition, devices and methods for attracting female mice. US provisional patent application (filed 17 August 2020; Patent App. Serial No. 63/066,716) (2020).

[74] R Core Team. R: A language and environment for statistical computing. (2019).

